# Biological Significance of NOTCH Signaling Strength

**DOI:** 10.3389/fcell.2021.652273

**Published:** 2021-03-26

**Authors:** Wei Shen, Jiaxin Huang, Yan Wang

**Affiliations:** ^1^Xiamen Cardiovascular Hospital, Xiamen University, Xiamen, China; ^2^Center for Structural Biology, School of Life Sciences and School of Medicine, Tsinghua University, Beijing, China

**Keywords:** Notch signaling, NICD, signaling strength, cell fate specification, development

## Abstract

The evolutionarily conserved NOTCH signaling displays pleotropic functions in almost every organ system with a simple signaling axis. Different from many other signaling pathways that can be amplified via kinase cascades, NOTCH signaling does not contain any intermediate to amplify signal. Thus, NOTCH signaling can be activated at distinct signaling strength levels, disruption of which leads to various developmental disorders. Here, we reviewed mechanisms establishing different NOTCH signaling strengths, developmental processes sensitive to NOTCH signaling strength perturbation, and transcriptional regulations influenced by NOTCH signaling strength changes. We hope this could add a new layer of diversity to explain the pleotropic functions of NOTCH signaling pathway.

## Introduction

Since the observation of notched wing in *Drosophila* and subsequent discovery of *notch* gene one century ago (Morgan, 1917), NOTCH signaling has been extensively studied. On/off switch of NOTCH signaling is found to play fundamental roles in cell differentiation, proliferation, and apoptosis across all species (Artavanis-Tsakonas et al., 1999; Harper et al., 2003; Lai, 2004; Lasky and Wu, 2005; Bolós et al., 2007; Penton et al., 2012). Surprisingly, the signaling axis of NOTCH is relatively simple despite its pleiotropic functions. Canonically, cell membrane-tethered NOTCH ligand binds to NOTCH receptor on the neighboring cell, which induces enzymatic cleavages of NOTCH receptor. The released notch intracellular domain (NICD) subsequently migrates into cell nucleus, where it binds with transcriptional factor CSL (CBF-1/RBP-J in mammal, Su(H) in *Drosophila*, and Lag-1 in *Caenorhabditis elegans*) together with other transcription co-factors to activate gene transcription (Kopan and Ilagan, 2009). Different from many other signaling pathways that contain kinase cascade-mediated signaling amplification processes, NOTCH signaling does not contain signaling intermediate to amplify the signal. In addition, NICD-CSL binding also triggers NICD ubiquitination that leads to its subsequent degradation (Fryer et al., 2004). Therefore, the scale and duration of gene transcription is sensitive to the dosage of NICD presented in cell nucleus.

Previous studies reviewed that protein level reduction caused by heterozygous mutation of NOTCH signaling components can lead to multiple developmental defects (Eldadah et al., 2001; McCright et al., 2002; Saito et al., 2003; Gale et al., 2004; Hozumi et al., 2004; McDaniell et al., 2006; Warthen et al., 2006; McKellar et al., 2007; Wu et al., 2007; Rubio-Aliaga et al., 2009; Hassed et al., 2012; Sargin et al., 2013; Meester et al., 2015; Southgate et al., 2015; Fischer-Zirnsak et al., 2019; Blackwood et al., 2020), suggesting developmental processes are sensitive to NOTCH signaling dosage. In addition, certain binary cell fate specifications are dependent on high/low regulation of NOTCH signaling strength instead of on/off switch of NOTCH signaling (Van de Walle et al., 2009, 2013; Gama-Norton et al., 2015), further highlighting the importance of NOTCH signaling strength regulation during development. Here, we reviewed the mechanisms of NOTCH signaling strength regulation; NOTCH components exhibiting haploinsufficiency and cell differentiation processes rely on precise NOTCH signaling strength. We hope this can add an extra layer of diversity to NOTCH signaling that plays pleotropic functions in almost every organ system with a simple signaling axis.

## Notch Signaling and Its Strength Regulation

### NOTCH Signaling Can Be Activated at Different Strength Levels Resulting in Distinct Transcriptional Responses

**FIGURE 1:**
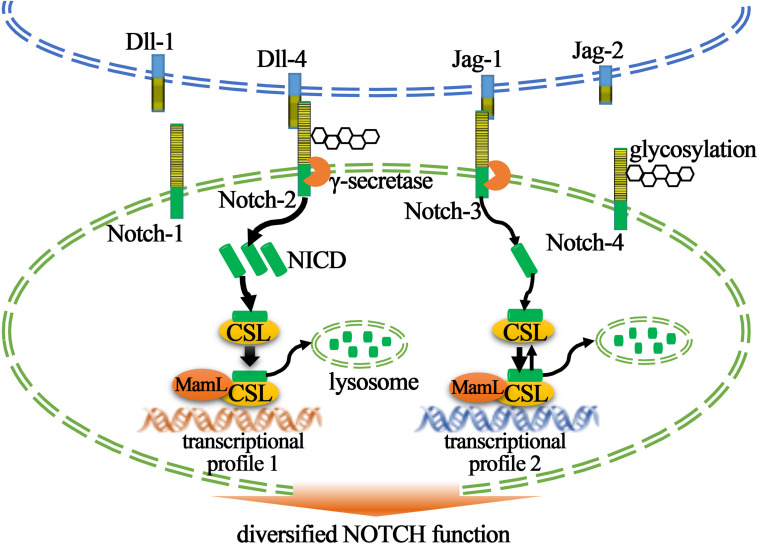
Cartoon illustrating NOTCH signaling and its strength regulations. Binding of NOTCH ligand (Jag-1, Jag-2, Dll-1, and Dll-4) to NOTCH receptor (Notch1, Notch-2, Notch-3, and Notch-4), a process that can be influenced by glycosylation of epidermal growth factor (EGF) domain (yellow stripes), triggers γ-secretase-mediated NOTCH receptor cleavage. Notch intracellular domain (NICD) is then freed and migrates into cell nucleus where it binds with transcriptional factor CSL, increases CSL dwell time on DNA, and recruits co-factor MamL to initiate the gene transcription. The ultimate transcription profile is affected by the dosage of NICD, which is also regulated by lysosome-mediated NICD degradation.

The mechanism of NOTCH signaling activation is highly conserved during evolution except for slight difference in terms of the number of NOTCH ligands and receptors across different species. In mammals, there are five NOTCH ligands (Dll-1, Dll-4, Jag-1, and Jag-2 are activators, and Dll-3 is an inhibitor) and four NOTCH receptors (Notch-1, Notch-2, Notch-3, and Notch-4), all of which contain extracellular epidermal growth factor (EGF)-like domains executing ligand–receptor binding. Subsequently after binding, NOTCH receptor undergoes two successive enzymatic cleavages mediated by ADAM10 and γ-secretase, releasing the NICD into cell nucleus where it binds with NOTCH signaling transcription factor CSL together with other co-factors to activate gene transcription (Figure 1). The most conserved direct targets of NOTCH signaling are basic helix-loop-helix (bHLH) transcription factors of hairy/enhancer of split (Hes) family and hairy/enhancer of split related with YRPW motif (Hey) family (Iso et al., 2003; Borggrefe and Oswald, 2009).

Unlike many other signaling pathways, NOTCH signaling can be activated at distinct strength levels due to following three reasons: (1) one NOTCH receptor can only release one NICD after ligand–receptor binding, (2) no signal intermediate or kinase cascade is involved to amplify the initial signal, and (3) NICD is subjected to proteasome-mediated degradation after transcriptional activation (Fryer et al., 2004). Since CSL and other transcriptional co-factors are always readily present in cell nucleus, the level of NICD generated determines the strength and duration of NOTCH signaling. A recent study by Nandagopal et al. (2018) showed that ligand intracellular domain (ICD) determined distinct membrane distribution pattern of Dll-4 (which is dispersed over membrane) and Dll-1 (which is clustered in puncta), which triggered sustained and pulsatile release of NICD, respectively. Interestingly, the two patterns of NICD release resulted in distinct downstream gene expression and consequently a different cell fate choice during embryonic myogenesis (Nandagopal et al., 2018). This confirmed gene transcription and cell fate specification can be influenced by NOTCH signaling strength perturbations.

### Mechanisms of NOTCH Signaling Strength Regulation

The frequency of NOTCH ligand and receptor binding determines the amount of NICD generated. In order to achieve a successful binding-induced NOTCH receptor cleavage, both ligand and receptor need to be presented on cell membrane in close proximity. Since all active NOTCH ligands and receptors are trans-membrane proteins that are subjected to consistent endocytosis, recycling, and degradation, the amount of NICD that could be potentially generated is affected by endocytic regulations of the ligands and receptors (Fortini and Bilder, 2009; Kandachar and Roegiers, 2012; Shen and Sun, 2020). Meanwhile, the four active NOTCH ligands (Jag-1, Jag-2, Dll-1, and Dll-4) in mammals exhibit different binding affinities (Benedito et al., 2009; Groot et al., 2014; Gama-Norton et al., 2015; Nandagopal et al., 2018), which further diversified the levels of NOTCH signaling strength in different cell contexts. The discovery of fringe glycosyltransferase also brought up the importance of EGF domain glycosylation, which can change ligand–receptor binding affinity and facilitate NOTCH receptor cleavage (Stanley and Okajima, 2010; Takeuchi and Haltiwanger, 2010; Kakuda and Haltiwanger, 2017). Thus, NOTCH signaling strength can be influenced by glycosylation of EGF domain in receptors. In addition to glycosylation, lipid composition of cell membrane can also influence NOTCH ligand–receptor binding via lipid–ligand interactions (Suckling et al., 2017). Underlying the binding affinity differences for different ligands, catch-bond that exhibited prolonged bond lifetimes upon tensile force application is shown to play important roles on modulating ligand–receptor binding (Luca et al., 2017). Collectively, the amount of ligand and receptor presented on cell membrane, the type of ligand binding to receptor, glycosylation of EGF domain, and lipid–ligand interaction all influence ligand–receptor binding and consequently the amount of NICD released.

The stability of NICD affects the duration of NOTCH signaling. NICD is generated following ligand–receptor binding and shuttles into cell nucleus, where it binds to transcription factor CSL together with co-factor of mastermind-like protein (MamL) and other chromatin modifiers to activate gene transcription. In addition to transcriptional regulations, CSL and MamL also recruit kinase CDK8 to phosphorylate NICD, which triggers protein ubiquitination on PEST (proline, glutamic acid, serine, and threonine-enriched) domain of NICD and proteasome-mediated NICD degradation (Fryer et al., 2004). Thus, NOTCH signaling is quickly turned down without re-supply of new NICD, which is a critical step to maintain proper levels of NOTCH signaling strength. Sustained NOTCH activation due to mutations in PEST domain can disrupt cell homeostasis and lead to various diseases, such as chronic lymphocytic leukemia (CCL; Ianni et al., 2009; Puente et al., 2011), marginal zone lymphoma (Trøen et al., 2008; Kiel et al., 2012), and increased proliferation of B-cell lymphoma cell (Zhang et al., 2014). Therefore, maintaining a proper level of NOTCH signaling strength is important.

## Developmental Processes Sensitive to Notch Signaling Strength Perturbation

### Haploinsufficiency of NOTCH Signaling Leads to Various Developmental Disorders and Diseases

Haploinsufficiency of NOTCH signaling components, a condition caused by heterozygous mutation producing only half amount of proteins, is related to the development of Adams–Oliver syndrome (AOS) characterized by congenital defects of limbs and scalp. *Notch-1* haploinsufficiency has been discovered in AOS patients with variable levels of cardiovascular anomalies, such as ventricular septal defects, aortic stenosis, regurgitation, and coarctation (Southgate et al., 2015). In line with the *Notch-1* haploinsufficiency discovered in AOS patients, another NOTCH signaling component *RBP-J* (coding the mammalian form of CSL) is also found to be haploinsufficient in AOS patients (Hassed et al., 2012), implying a causative role of reduced NOTCH signaling strength during the development of AOS. In addition, Dll-4 is likely the NOTCH ligand responsible for generating the proper level of NOTCH signaling in this case, as AOS patients are also reported to carry *Dll-4* heterozygous mutation (Meester et al., 2015). Collectively, *Dll-4*, *Notch-1*, and *RBP-J* haploinsufficiencies are associated with AOS.

Haploinsufficiency of NOTCH signaling components also leads to the development of Alagille syndrome (AGS) that affects multiple organs including the liver, heart, eye, kidney, and bone. AGS patients are reported to carry heterozygous mutation of *Jag-1* (Warthen et al., 2006) or *Notch-2* (McDaniell et al., 2006) or both (Brennan and Kesavan, 2017). Mice models with heterozygous mutations of *Jag-1* and *Notch-2* also successfully recapitulated certain symptoms of AGS (McCright et al., 2002), suggesting that reduction of NOTCH signaling leads to the development of AGS. Interestingly, some AGS-related defects can also be associated with NOTCH signaling components’ haploinsufficiency independent of AGS. For example, tetralogy of Fallot, a congenital heart disease frequently observed in AGS patients, can be found patients carrying *Jag-1* heterozygous mutation without diagnosis of AGS (Eldadah et al., 2001; Bauer et al., 2010). Another congenital heart disease named bicuspid aortic valve, a condition lacking one valve between the left ventricle and main artery, is associated with *Notch-1* haploinsufficiency and also independent of AGS (McKellar et al., 2007). Therefore, NOTCH signaling haploinsufficiency can lead to the development of AGS and multiple cardiovascular defects.

Beyond above diseases, systematic examination of mice models also showed that NOTCH signaling haploinsufficiency can disrupt the development of many organs and tissues where NOTCH signaling plays essential functions. In addition to the reported AGS symptoms (Huppert, 2016), *Jag-1^±^* mice also showed degeneration of ganglionic eminence caused by suppressed neuronal progenitor cell proliferation (Blackwood et al., 2020) and spatial memory impairment (Sargin et al., 2013). *Dll-4^±^* mice displayed various artery defects including failed remodeling of yolk sac vasculature, artery regression, artery stenosis, atresia aorta, and defected arterial branching (Gale et al., 2004), consistent with the essential function of *Dll-4* during artery development (Shutter et al., 2000; Cristofaro et al., 2013). *Dll-1^±^* mice on the other hand displayed smaller bodies, reduced cholesterol and triglyceride levels, suppressed immune system, bradycardia (Rubio-Aliaga et al., 2009), and brain disorders (Fischer-Zirnsak et al., 2019). Collectively, haploinsufficiency of NOTCH signaling components can lead to various developmental disorders.

### NOTCH Signaling Strength in Arterial Endothelial Cells

The change of NOTCH signaling strength influences artery endothelial cell fate (vs. hematopoietic stem cell fate) specification and artery branching (vs. elongation). During early embryonic development, progenitor cells resident in aorta-gonad-mesonephros (AGM) region can give rise to either arterial endothelial cell or hematopoietic stem cell (HSC) depending on NOTCH signaling strength. *In vivo* study found that Dll-4 activated high NOTCH signaling while Jag-1 activated low NOTCH signaling in AGM region, where high NOTCH signaling specifies endothelial cell fates while low NOTCH signaling specifies HSC fates. Neutralization of Dll-4 via antibody blockage lowered NOTCH activity and forced AGM cells to differentiate into HSCs, suggesting a NOTCH-strength-dependent cell fate specification (Gama-Norton et al., 2015). In addition, NOTCH signaling strength also influences branching of the artery during the generation of vascular network. New tip cell specification, a requirement for artery branching, is inhibited by Dll-4-activated high NOTCH signaling while requires Jag-1-activated low NOTCH signaling. *Jag-1* mutated mice, in which Dll-4 dominated in the artery and activated high NOTCH signaling, exhibited significantly reduced vascular branching. In contrast, overexpression of Jag-1 outcompeted Dll-4 and significantly promoted vascular branching. During the establishment of this signaling strength difference, fringe-mediated glycosylation of NOTCH played a critical role on potentiating Dll-4-activated high NOTCH signaling and suppressing Jag-1-mediated low NOTCH signaling. Removing fringe suppressed Dll-4-activated high NOTCH signaling and permitted Jag-1-activated low NOTCH signaling, resulting in enhanced vascular branching (Benedito et al., 2009).

Further study showed that the developmental difference resulting from Dll-4- and Jag-1-activated NOTCH signaling during vascular branching was purely due to the strength difference of NOTCH signaling. Administration of γ-secretase inhibitor (DAPT) into *Jag-1*-mutated mice lowered Dll-4-activated NOTCH signaling and rescued vascular branching defects (Benedito et al., 2009). Therefore, it is the strength of NOTCH signaling that determines the artery branching. Utilizing hybrid proteins created by swapping the ICD of Jag-1 and Dll-4 found that ICDs of NOTCH ligands determine the potential of NOTCH signaling strength that could be activated by Jag-1 or Dll-4. Cytoskeletal filament vimentin specifically binds to Jag-1 ICD and determines the pulling force critical for binding-induced NOTCH receptor cleavage. Hybrid protein fused by Jag1 ICD and Dll-4 extracellular domain (ECD) generated NOTCH signaling resembling ligand Jag-1 (Antfolk et al., 2017). Collectively, different NOTCH ligands hold distinct capacities to activate NOTCH signaling, and ligand ICDs play fundamental roles on influencing the activation potential.

### NOTCH Signaling Strength in Vascular Smooth Muscle Cell

NOTCH signaling is required for vascular smooth muscle cell (VSMC) differentiation. VSMCs are recruited to vascular endothelial cells during vasculogenesis and play crucial roles on maintaining normal vascular tone in response to hemodynamic changes, especially in arteries where multiple layers of VSMCs are attached to endothelial cells (Zhuge et al., 2020). During vasculogenesis, VSMC progenitors are recruited to vascular bed where endothelial cell expresses Jag-1-activated NOTCH signaling in VSMC progenitor cells, resulting in expression of smooth cell markers of α*-SMA* and *SM-22*α and the final specification of VSMC fate (Noseda et al., 2006; High et al., 2008). Meanwhile, the activated NOTCH signaling in VSMCs also directly activated *Jag-1* expression in the newly formed VSMCs, allowing further propagation of NOTCH signaling in the outer layers of VSMCs (Hoglund and Majesky, 2012; Manderfield et al., 2012; van Engeland et al., 2019). Therefore, Jag-1-activated NOTCH signaling is essential to maintain VSMC fate and form a multiple-layer structure of VSMCs in the artery.

Elevated NOTCH signaling strength triggered the proliferation of VSMCs. Unlike skeletal muscle cells or cardiomyocytes, both of which are terminally differentiated and quiescent, VSMCs holds the ability to proliferate, dedifferentiate, and even transdifferentiate into macrophage-like cells in response to vascular injury or environmental stimulus (Bennett et al., 2016; Basatemur et al., 2019; Zhuge et al., 2020). The elevated NOTCH signaling has been observed in arterial injury (Wang et al., 2002) and atherosclerotic lesions (Davis-Knowlton et al., 2019), both of which involve VSMC proliferation. An *in vitro* study also showed that over-expression of NICD in VSMCs can increase cell proliferation (Sweeney et al., 2004), which seems contradictory to the fact that NOTCH signaling promotes the quiescent status of VSMCs characterized by the expression of α-SMA. However, NICD over-expression generally resulted in sustained high NOTCH signaling, and detailed *in vitro* studies clarified that NOTCH downstream targets of Hey are promoted under this condition and are responsible for increased VSMC proliferation and suppressed α-SMA expression via negative feedback loops. Specifically, the increased Hey, a transcriptional repressor, can in term prevent the transcription of α*-SMA* through directly binding to α*-SMA* promoter (Tang et al., 2008). Meanwhile, Hey also inhibits the expression of cyclin-dependent kinase inhibitor *P27^*kip*1^*, allowing re-entering into the cell cycle (Havrda et al., 2006). Conclusively, NOTCH signaling is required for expression of VSMC markers to maintain VSMC fate, while elevated NOTCH signaling can suppress VSMC marker expression and promote VSMC proliferation.

### NOTCH Signaling Strength in T-Cell Lineage

NOTCH signaling strength determines αβ T-cell (vs. γδ T-cell) specification in T-cell lineage. Postnatal development of T immune cell in the thymus requires activation of NOTCH signaling in the hematopoietic progenitor cells (HPCs) that migrated from the bone marrow. NOTCH signaling activation inhibits non-T-cell cells including myeloid lineage during early stages and B-cell during late stages (Wilson et al., 2001). However, after T-cell fate is committed, the strength of NOTCH in T-cell lineage determines T-cell sub-lineage specifications between αβ T-cell and γδ T-cell. An *in vitro* study showed that human OP9-Dll1/4 that served as NOTCH signal-sending cell can stimulate the differentiation of human HPCs into T-cells populated with both αβ T-cell and γδ T-cell. Interestingly, lowering NOTCH signaling strength via adding a series of γ-secretase inhibitor (DAPT) with increasing concentrations gradually switched γδ T-cell into αβ T-cell (Van de Walle et al., 2009), documenting NOTCH-strength-dependent cell fate determination between the two T-cell subtypes.

The strength changes of NOTCH signaling in T-cell lineage is caused by binding with different NOTCH ligands that hold distinct receptor binding affinities. Still in human HPCs, Jag-2 exhibited strong NOTCH activation potential and directed HPCs predominantly into γδ T-cell (Van de Walle et al., 2011, 2013); Dll-4 instead induced a relatively weak NOTCH signaling and generated both γδ T-cell and αβ T-cell, while Jag-1 induced the weakest NOTCH signaling and generated mainly αβ T-cell (Van de Walle et al., 2013). Collectively, the diverted expression of NOTCH ligands maintained a diverse range of NOTCH signaling strength, which balanced the population of αβ T-cell and γδ T-cell. Surprisingly, mice HPCs also utilize the strength difference of NOTCH signaling to determine the αβ T-cell fate and γδ T-cell fate but in an opposite way that low NOTCH signaling favors γδ T-cell (Washburn et al., 1997). It is intriguing how this difference between human and mouse is generated, while the NOTCH-strength-dependent cell fate determination is indeed conserved during evolution.

### NOTCH Signaling Strength in Marginal Zone B Cell

Differentiation of marginal zone B (MZB) cell, an immune cell developed in marginal zone of the spleen, relies on NOTCH signaling dosage. Notch-2 is preferentially expressed in B-cell and is prominent in splenic marginal zone, suggesting its potential function on MZB cell differentiation. Homozygous mutation of *Notch-2* completely eliminated MZB cells. Interestingly, heterozygous mutation of *Notch-2*, in which 50% of *Notch-2* mRNA still expressed, resulted in partial reduction of MZB cells (Saito et al., 2003). Consistent with *Notch-2* mutant, mutational study of *MamL-1* (transcriptional co-factor of CSL) showed a similar dose-dependent regulation of MZB cell differentiation. Wild-type, heterozygous mutation of *MamL-1* and homozygous mutation of *MamL-1* showed sequential reduction of MZB cell number (Wu et al., 2007), suggesting all these defects are due to reduction of NOTCH signaling strength.

Other studies on NOTCH ligands found that it is the ligand of Dll-1 that activates Notch-2 for MZB cell differentiation in the spleen. Mutational study of *Dll-1* similarly showed a sequential reduction of MZB cell among wild-type, heterozygous mutation of *Dll-1* and homozygous mutation of *Dll-1* (Hozumi et al., 2004). A later study in splenic stromal cells further confirmed that Dll-1 expressed by these cells are responsible for activating NOTCH signaling required for MZB cell differentiation (Fasnacht et al., 2014). Collectively, these comparison studies on heterozygous mutation of *Notch-2*, *MamL-1*, and *Dll-1* suggest that MZB cell differentiation is dependent on NOTCH dosage. MZB cell differentiation likely requires NOTCH signaling to be above certain threshold. Haploinsufficiency of these NOTCH signaling components made many cells fail to reach this threshold and resulted in big reduction of mature MZB cells.

### NOTCH Signaling Strength in Pancreatic Progenitor Cells

Stepwise downregulation of NOTCH signaling strength in pancreatic endocrine progenitor cells drives the transition from quiescence to proliferation and from proliferation to differentiation. NOTCH signaling is well-known for its function on maintaining pancreatic progenitor cells, and suppressing NOTCH signaling triggered the progenitor cells to differentiate into pancreatic secreting cells (Apelqvist et al., 1999). Interestingly, lineage tracing observation of pancreas development in *zebrafish* discovered a stepwise downregulation of NOTCH signaling strength in quiescent endocrine progenitor cells, proliferating endocrine progenitor cells and differentiated mature endocrine cells. Lowering NOTCH signaling strength via applying low concentration of γ-secretase inhibitor (DAPT) to the developing pancreas promoted progenitor cell proliferation and consequently expanded pancreatic endocrine progenitor pool. However, saturated DAPT led to differentiation of pancreatic secreting cells accompanied by drastically reduced progenitor pool (Ninov et al., 2012), confirming moderate NOTCH signaling strength is required for the proliferation of pancreatic endocrine progenitor cells.

Conclusively, high NOTCH signaling strength maintains quiescent state of pancreatic endocrine progenitor cell, moderate NOTCH signaling strength triggers its proliferation, and low NOTCH signaling strength leads to its final differentiation. Combining NOTCH haploinsufficiency-related disorders, precise strength requirements in artery endothelial cell, VSMC, T-cell, and MZB cell, developmental processes can be sensitive to NOTCH signaling strength perturbations (summarized in Table 1). Therefore, re-examining NOTCH-regulated processes with consideration of signaling strength would likely offer new insights to explain the pleotropic functions of NOTCH.

**TABLE 1:** Developmental processes sensitive to NOTCH signaling strength.

NOTCH changes	Phenotypic changes	Study species	Reference paper
*Jag-1* haploinsufficiency	Alagille syndrome	Human	Warthen et al., 2006; Brennan and Kesavan, 2017
	Alagille syndrome	Mouse	McCright et al., 2002; Huppert, 2016
	Tetralogy of Fallot	Human	Eldadah et al., 2001; Bauer et al., 2010
	Brain malfunctions	Mouse	Sargin et al., 2013
	Neuron stem cell reduction	Mouse	Blackwood et al., 2020
*Dll-1* haploinsufficiency	Brain malfunctions	Mouse	Fischer-Zirnsak et al., 2019
	Growth retardation and metabolic disorder	Mouse	Rubio-Aliaga et al., 2009
*Dll-4* haploinsufficiency	Adams–Oliver syndrome	Human	Meester et al., 2015
	Broad artery defects	Mouse	Gale et al., 2004
*Notch-1* haploinsufficiency	Adams–Oliver syndrome	Human	Southgate et al., 2015
	Bicuspid aortic valve	Human	McKellar et al., 2007
*Notch-2* haploinsufficiency	Alagille syndrome	Human	McDaniell et al., 2006; Brennan and Kesavan, 2017
	Alagille syndrome	Mouse	McCright et al., 2002
*RBP-J* haploinsufficiency	Adams–Oliver syndrome	Human	Hassed et al., 2012
High NOTCH signal	AGM cell differentiates to endothelial cell	Mouse	Gama-Norton et al., 2015
Low NOTCH signal	AGM cell differentiates to hematopoietic stem cell		
High NOTCH signal	Inhibit artery tip cell specification and artery branching	Mouse	Benedito et al., 2009; Antfolk et al., 2017
Low NOTCH signal	Promote artery tip cell specification and artery branching		
High NOTCH signal	VSMC in proliferative state	Human, rat, mouse	Havrda et al., 2006; Tang et al., 2008
Moderate NOTCH signal	VSMC in contractile and quiescent state		Noseda et al., 2006; High et al., 2008
High NOTCH signal	Human HPC differentiates into γδ T-cell	Human	Van de Walle et al., 2013
Low NOTCH signal	Human HPC differentiates into αβ T-cell		
High NOTCH signal	Mouse HPC differentiates into αβ T-cell	Mouse	Washburn et al., 1997
Low NOTCH signal	Mouse HPC differentiates into γδ T-cell		
High NOTCH signal	Normal MZB cell differentiation	Mouse	Saito et al., 2003; Hozumi et al., 2004; Wu et al., 2007; Fasnacht et al., 2014
Moderate NOTCH signal	Reduced MZB cell differentiation		
No NOTCH signal	No MZB cell differentiation		
High NOTCH signal	Pancreatic progenitor in quiescent	Zebrafish	Ninov et al., 2012
Moderate NOTCH signal	Pancreatic progenitor in proliferation		
Low NOTCH signal	Pancreatic progenitor in differentiation		

## Mechanisms Underlying Distinct Transcriptional Activation by Different Strengths of Notch Signaling

Different duration and dynamics of NOTCH signaling strengths yield distinct transcriptional responses, which resulted in distinct developmental consequences. A new study reported by Nandagopal et al. (2018) showed that the conserved NOTCH direct target genes of *Hes1* and *Hey1/L* responded differently to the level changes of NICD, the active component of NOTCH signaling. Interestingly, *Hes1* and *Hey1/L* did not simply adjust their expression levels in proportion to the amount of NICD generated. High and sustained NOTCH activation induced by Dll-4 initiated *Hey1/L* transcription compared to pulsatile NOTCH activation induced by Dll-1, instead of proportionally increasing *Hes1* that was already transcribed under pulsatile NOTCH activation (Nandagopal et al., 2018). Considering NICD pattern change alone can initiate new gene expression, and it is likely that level changes of NICD would mediate chromatin modifications for gene transcription.

Dosage change of NICD can influence CSL-DNA binding kinetics, NICD dimerization, and chromatin opening. A recent study using live cell imaging changed the classical model that CSL stays on chromatin to repress gene transcription until the arrival of NICD that switches CSL into a transcriptional activator (Morel et al., 2001; Borggrefe and Oswald, 2009). Instead, CSL consistently binds to and detaches from DNA, and NICD along or together with co-factor of MamL can change the binding dynamics of CSL onto DNA by increasing its chromatin dwell time and potentially the binding loci (Gomez-Lamarca et al., 2018). NICD dimerization, utilizing two NICDs for gene transcription, is essential for certain NOTCH downstream gene expression. Suppressing of NICD dimerization leads to various developmental disorders including heart anomalies and defective MZB cell differentiation; therefore, shifted balance between dimerized NICD and monomer NICD that can be influenced by NICD dosage is going to affect transcriptional profile as well (Hass et al., 2015; Kobia et al., 2020). The changing of gene transcription under different NICD dosages can be further proven by the observation that NICD can change chromatin structure (Gomez-Lamarca et al., 2018), opening of which permits more gene transcription. Collectively, NICD not only switches CSL into transcription activator but also modulates the dynamics of CSL-DNA binding, NICD dimerization, and chromatin structure, thus affecting gene transcription both qualitatively and quantitatively.

## Conclusion

The on/off switch of NOTCH signaling is well-recognized for its function on regulating cell differentiation, proliferation, and apoptosis. The pleotropic function of NOTCH signaling seems contradictory to the simple setting of NOTCH signaling axis. Genetic studies discovered heterozygous mutations of NOTCH signaling components in various developmental disorders and diseases, suggesting that organ development is also sensitive to NOTCH signaling dosage. More importantly, simple changes of NOTCH signaling strength influences the binary cell fate determinations and cell proliferation and differentiation during artery, postnatal T-cell, MZB cell, and pancreas development, suggesting that NOTCH signaling strength changes can be as important as on/off switch of the signaling. Underlying NOTCH signaling strength changes and NICD–CSL complex-mediated gene transcriptions are changed both quantitatively and qualitatively to direct distinct cellular responses. Therefore, NOTCH signaling strength can add a new layer of diversity to explain the pleotropic functions of NOTCH.

## Author Contributions

WS and YW conceived the study. All authors contributed their expertise in NOTCH signaling, epigenetic regulation, and cardiovascular development, respectively and contributed to the finalization of this manuscript significantly.

## Conflict of Interest

The authors declare that the research was conducted in the absence of any commercial or financial relationships that could be construed as a potential conflict of interest.
